# Interplay between Cytokine Circuitry and Transcriptional Regulation Shaping Helper T Cell Pathogenicity and Plasticity in Inflammatory Bowel Disease

**DOI:** 10.3390/ijms21093379

**Published:** 2020-05-11

**Authors:** Shin-Huei Fu, Ming-Wei Chien, Chao-Yuan Hsu, Yu-Wen Liu, Huey-Kang Sytwu

**Affiliations:** 1 National Institute of Infectious Disease and Vaccinology, National Health Research Institutes, No. 35, Keyan Road, Zhunan Town, Miaoli County 35053, Taiwan; winniefold@gmail.com (S.-H.F.); chienmw0103@hotmail.com (M.-W.C.); hsu.chaoyuan@gmail.com (C.-Y.H.); 2 Department and Graduate Institute of Microbiology and Immunology, National Defense Medical Center, No. 161, Section 6, Minquan East Road, Neihu District, Taipei 11490, Taiwan; 3 Molecular Cell Biology, Taiwan International Graduate Program, Academia Sinica, No. 128, Academia Road, Section 2, Nankang, Taipei 11529, Taiwan; candy_77615@yahoo.com.tw; 4 Graduate Institute of Life Sciences, National Defense Medical Center, No. 161, Section 6, Minquan East Road, Neihu District, Taipei 11490, Taiwan

**Keywords:** T helper cells, cytokines, transcription factors, plasticity, transdifferentiation, conversion, inflammatory bowel diseases, Crohn’s disease, ulcerative colitis

## Abstract

Inflammatory bowel disease (IBD) is a chronic disorder manifested as Crohn’s disease (CD) and ulcerative colitis (UC) characterized by intestinal inflammation and involves a dysregulated immune response against commensal microbiota through the activation of CD4 T helper cells. T helper cell differentiation to effector or regulatory phenotypes is controlled by cytokine networks and transcriptional regulators. Distinct polarized T helper cells are able to alter their phenotypes to adapt to diverse and fluctuating physiological environments. T helper cells exhibit intrinsic instability and flexibility to express cytokines of other lineages or transdifferentiate from one T helper cell type to another in response to various perturbations from physiological cytokine milieu as a means of promoting local immunity in response to injury or ensure tissue homeostasis. Furthermore, functional plasticity and diversity of T helper cells are associated with pathogenicity and are critical for immune homeostasis and prevention of autoimmunity. In this review, we provide deeper insights into the combinatorial extrinsic and intrinsic signals that control plasticity and transdifferentiation of T helper cells and also highlight the potential of exploiting the genetic reprogramming plasticity of T helper cells in the treatment of IBD.

## 1. Introduction to Inflammatory Bowel Disease

### 1.1. Genetic Predisposition

Inflammatory bowel disease (IBD) is caused by the interplay of genetic susceptibility, environmental factors, and uncontrolled immune responses [1]. Crohn’s disease (CD) and ulcerative colitis (UC) are two major forms of IBD and present inflammatory profiles associated with complex etiopathogenic factors, including dysfunction of the intestinal immune system and disruption of intestinal epithelial barrier integrity [2]. Although the exact etiology of IBD is still not completely clear, various genetic abnormalities and genetic loci have been associated with both forms of IBD, leading to dysregulated T cell responses to commensal bacteria based on research in animal models and human genetics [3]. Following the first report of association of *NOD2* gene variants in CD [4], genome-wide association studies identified 242 associated genomic loci containing susceptibility genes for CD, UC, or both [5,6], providing insights into their pathogenic mechanisms. Among these single nucleotide polymorphisms, an exceptional proportion of these exhibited pathophysiologically relevant associations, with mutations implicated in T cell response, T cell activation, and immunosuppression [5]. Variants in *IL23R*, *IL12B*, *JAK2*, *STAT3*, *STAT4*, *PRDM1*, and *IKZF1* were identified in both UC and CD, implying an important role of T helper (Th)1/Th17 and interleukin (IL)-12/IL-23 pathways toward the pathogenesis of IBD [7,8,9]. Other susceptibility genes that regulate transforming growth factor (TGF)-β ignaling (*SMAD3*, *SMAD7* and *SMURF*) were also associated with IBD [10,11]. Importantly, the CD-specific risk variants identified related to the immune system include *PTPN22*, *IL2RA*, *IL27*, and *TNFSF11* [12], whereas *PTPN22* appears to protect against UC. Defects in immunosuppressive cytokine IL-10 were also associated with CD and UC, while loss-of-function mutations in IL-10 receptor subunit (*IL10RA* and *IL10RB*) genes caused an infantile-onset, CD-like form of enterocolitis [13,14,15]. Further studies of IBD susceptibility gene dysfunctions in tissue cultures and animal models are required to elucidate their contributions to disease risk and accelerate translational benefits of developing new treatments for IBD patients [16].

### 1.2. CD4^+^ T Cells

**Figure 1:**
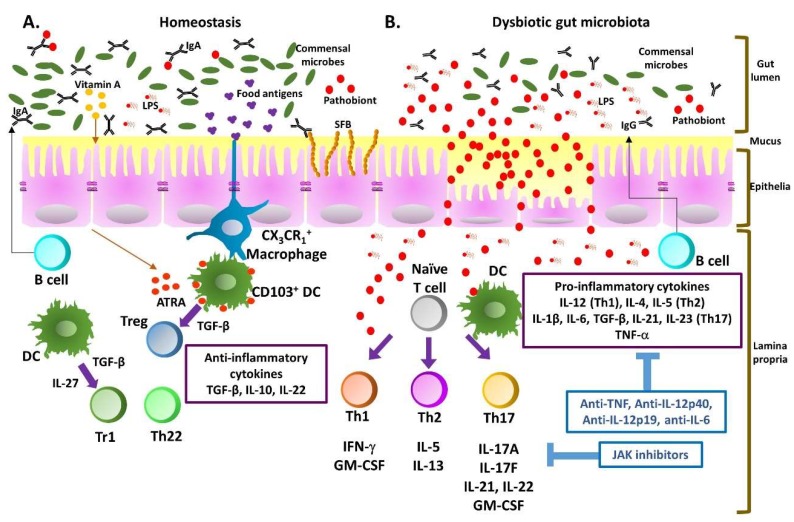
Dynamics of effector T cell development and potential of therapeutic targets during inflammatory bowel disease (IBD) progression. (**A**) During homeostasis, a symbiotic gut microbiota leads to the development of a functional barrier promoting the development of tolerogenic immune cell responses to the microbiota. Foxp3^+^ regulatory T cells (Tregs), FoxP3-negative type 1 regulatory T cells (Tr1), and Th22 dominantly appear in the gut lamina propria (LP) and produce transforming growth factor (TGF)-β, IL-10, and IL-22, leading to T cell regulation and epithelial cell maintenance/protection at homeostasis; (**B**) Pathobiont overgrowth leads to the loss of barrier integrity and the increase of intestinal permeability, allowing immune cell infiltration of the gut LP. Translocation of bacteria and bacterial components trigger the intestinal immune system to direct a harmful proinflammatory response, promoting Th1, Th2, and Th17 responses by dendritic cells (DC). Meanwhile, IL-12 leads to Th1 polarization, IL-4 and IL-5 lead to Th2 differentiation, and IL-6, IL-1β, and IL-21 in addition to TGF-β secretion differentiate naïve T cells into Th17. Tumor necrosis factor- (TNF-α) is a key inflammatory mediator produced by immune cells and leads to accelerated inflammation. Therapies targeting the initiation and progression phases of disease are indicated in blue.

During perturbation of intestinal epithelial barrier integrity due to either mucosal susceptibility or defects in areas of the gut lumen, microbial translocation occurs, triggering activation of innate immune response in lamina propria. Antigen-presenting cells then mediate the differentiation of naïve T cells into effector Th cells, which alter gut homeostasis. Of interest, many environmental and inflammatory factors associated with IBD pathology, such as pro-inflammatory mediators, cytokines, microbiome dysbiosis, metabolism, and diet, are linked to the development of pathogenic T cells. Although the etiology of IBD remains unknown, the development of chronic intestinal inflammation occurs when uncontrolled inflammatory and memory-phenotype CD4^+^ T cells infiltrate the lamina propria of IBD patients due to barrier dysfunction and loss of immune tolerance to intestinal antigens [1]. Therefore, a delicate balance between regulatory and inflammatory CD4^+^ T cells is pivotal to prevent failure of homeostasis and subsequent tissue damage in the intestine. Triggered by environmental cytokine milieu, naive CD4^+^ T cells differentiate into functionally distinct helper subsets characterized by a specific network of transcriptional regulators and unique cytokine profiles mounted against various invading pathogens. CD4^+^ T cells can differentiate into effector (Th1, Th2, Th17, and follicular Th (Tfh) cells) or regulatory phenotypes (forkhead box protein (Foxp)3^+^ regulatory T cells (Tregs) and type 1 regulatory T cells (Tr1)); further, these cytokine-producing Th subsets including Th1 (interferon (IFN)-γ), Th2 (IL-4, IL-5, IL-13), Th9 (IL-9), Th17 (IL-17), Th22 (IL-22), Treg (IL-10), and Tr1 (IL-10) cells, which are all involved in the development of IBD. Classically, dysregulated mucosal immunity in CD is thought to be a Th1-driven disease, whereas UC is thought to be a Th2-mediated response. Recent studies highlighted the critical role of the newly identified Th17 subset in IBD as an important mediator of host defense and homeostasis in the intestine [17,18,19,20]. Since segmented filamentous bacteria (SFB) were identified to induce intestinal Th17 cells [21], emerging data indicate that the aberrant expansion of SFB in the gut, a previously unidentified specific form of small intestinal bacterial overgrowth found in about 25% of patients with CD, could play a role in the pathogenesis of chronic intestinal inflammatory diseases through persistent activation of Th17 cells [22]. Cytokine expression patterns toward Th1 and Th17 profiles were identified in the colons of both CD and UC patients, while Th2 responses distinguished UC from CD [23,24,25]. Moreover, the percentages of mucosal IL-17-producing Th17, IFN-γ, and IL-17 double-producing Th1/Th17 cells were increased in patients with IBD [26]. The roles of IL-22 and IL-17A/IL-23 axis were also implicated in the inflamed mucosa of patients and associated with disease activity in both UC and CD [27,28,29]; a similar pattern of IBD disease extent was observed in mouse models. Transfer of CD4^+^CD45RB^hi^ T cells from normal donors to immunocompromised mice led to IBD-like syndrome with an aberrant Th1 response and excess production of IFN-γ and tumor necrosis factor (TNF)-α, demonstrating an association with tissue injury [30]. In addition, both Th1 cells and Thl7 cells were shown to be involved in 2,4,6-trinitrobenzenesulphonic acid (TNBS)-induced IBD [31,32], while dextran sulfate sodium salt (DSS) colitis was shown to switch from a Th1-Th17-mediated acute inflammation to a Th2-dominant immune response in the chronic state [33]. Furthermore, diminished or dysfunctional regulatory T cells defined by their ability to produce immunosuppressive cytokines such as IL-10 and transforming growth factor (TGF)-β could be involved in disturbed mucosal homeostasis of IBD [34]. Moreover, increased Th1, Th2, and Th17 responses and reduced Treg and Tr1 responses were suggested to play roles in IBD pathophysiology [3,17,35]. Therapeutic development to target the aberrance of Th responses and lack of activation and expansion of regulatory T cells holds promise for the treatment of IBD (Figure 1).

### 1.3. Transcriptional Regulation

The process of differentiation is controlled by networks of master and accessory transcription factors that instruct the expression of signature cytokines and determine the effector functions of T helper cells. Based on their transcription factor expression, CD4^+^ T helper cells can be subdivided into different lineages: Th1 (which express transcription factor T-box-expressed-in-T-cells (T-bet)), Th2 (which express GATA-binding protein 3 (GATA-3)), Th17 (which express retinoic acid-related orphan receptor (ROR)γt in mice and ROR-C in humans), Tfh (which express B cell lymphoma (Bcl)-6), and Treg cells (which express Foxp3). Since Th17 cells and Th1/17 cells were identified as the pathogenic cells in IBD, the blockage of RORγt using a selective RORγt inverse agonist demonstrated a promising pharmacological profile of decreased Th17 and Th1/17 populations in the mesenteric lymph node in a murine colitis model [36]. Except for master transcription factors, suppressors of cytokine signaling (SOCS) proteins directly regulate the activation of signal transducers and activators of transcription (STATs) by binding to cytokine receptors and associated janus kinase (JAK) proteins to orchestrate the polarization of CD4^+^ T cells [37]. IL-12 promotes Th1 cell polarization through activation of STAT4, while IL-23 activates STAT3 to advance Th17 cell amplification [38]. The involvement of STAT3 regulates T cell responses in opposing directions in response to the pro-inflammatory cytokine IL-6 and the anti-inflammatory cytokine IL-10 [39]. Recently, the transcription factor Bach2 was shown to serve as a highly conserved repressor, which is critical for the formation and function of CD4^+^ T cell lineages (Th1, Th2, Th9, Th17, Tfh, and Treg cells) and the correlation with immune-mediated diseases [40]. However, increasing evidence regarding conversion among polarized T helper cells indicated that CD4^+^ T cells are far from stable and terminally differentiated lineages but more plastic than initially thought [41]. Recent findings demonstrated that eomesodermin (Eomes), a transcription factor of the T-box family, plays a critical role in Th plasticity by shifting the Th17 phenotype toward non-classic Th1 cells; on the contrary, Eomes is a lineage-specifying transcription factor for human IL-10 and IFN-γ co-producing Tr1 cells [42,43]. It is of particular importance to understand the molecular mechanisms regulating T cell lineage specification and plasticity to highlight the potential of genetic plasticity exploitation of Th cells in the treatment of IBD.

### 1.4. Cytokine-Mediated Regulation

Cytokines of the intestinal microenvironment dominate immunological responses after mucosal damage and dictate homeostatic or pro-inflammatory pathways in IBD. Abnormalities in the expression of cytokines such as IL-2, IL-4, IL-10, and IFN-γ serve as key factors in the dysregulation of intestinal immunity associated with pathological processes [44]. Data from clinical trials and experimental models of IBD implicated T cell-derived cytokines as pivotal mediators of intestinal insults. TGF-β, IL-6, IL-23, IL-1β, and IL-21-driven differentiation of Th17 cells promotes the expression of effector cytokines IL-17A, IL-17F, IL-21, and IL-22 with both tissue-protective and inflammatory effects in the gut. IL-21, a crucial component of the inflammatory process in the gut, is highly expressed in CD and is essential for sustaining the Th1-mediated immune response and the amplification and stabilization of the Th17 phenotype [45]. Previous studies suggested that the pathogenicity of Th17 cells is determined by IL-23 and TGF-β3 in the local micro-environment, with important roles identified for commensal microbiota and aryl hydrocarbon receptor (Ahr) ligands in stabilizing Th17 gene expression in vivo, linking environmental cues to Th cell differentiation [46]. Th17 cells were shown to exhibit flexible programming of effector functions in response to environmental stimuli and convert into Th1-producing cells [47]. In addition to T cell signature cytokines, inflammatory cytokines such as IL-6, IL-12, IL-23, and IL-27 secreted from activated macrophages are thought to contribute to differentiated CD4^+^ T lineage and the pathogenesis of IBD [48,49,50]. The relationship between IL-12, IL-23, and heterodimeric cytokines sharing the common p40 subunit may play a major role in the generation of pathogenic Th1 and Th17 cells in IBD [51,52]. IL-23 amplifies the Th17 cell response and restrains the regulatory T cell response, favoring gut inflammation and implicating the IL-23/IL-17 axis in autoimmune inflammation [53,54,55,56]. The efficacy of targeting IL-12p40 in mouse colitis models and treatment of monoclonal antibodies (ustekinumab and briakinumab) targeting p40 in IBD patients suggest that blocking IL-23 signaling is a promising therapeutic approach for IBD [38,57,58,59].

Several immunoregulatory abnormalities have been reported in patients with IBD, including imbalance between pro-inflammatory and immunoregulatory cytokines. Negative regulatory molecules such as TGF-β and IL-10 seem to be important in downregulating tissue-damaging T cell responses in the gut mucosa [36]. The differentiation of inflammatory Th17 cells and suppressive Treg subsets is reciprocally regulated by relative concentrations of TGF-β [56]. IL-10 is strongly immunosuppressive by inhibiting both antigen presentation and production of proinflammatory cytokines such as IL-1, TNF-α, IFN-γ, and IL-6, thereby leading to attenuation of intestinal inflammation. IL-10-deficient mice developed spontaneous colitis and enteritis and patients with mutations in IL-10 or its receptor, IL-10R, showed increased susceptibility to IBD [13,14,15], while IL-10R signaling was shown to regulate Th17 polarization and T cell proliferation in infantile-onset IBD patients [60,61,62,63]. In addition, elevated IL-10 mRNA expression in the mucosa of patients with both UC and CD suggested that IL-10 is a crucial regulatory cytokine for controlling the inflammatory response in IBD [44,64,65]. A highly significant increase in IL-10 mRNA levels in T lymphocytes and an increased frequency of IL-10-positive cells were observed in the colons of UC patients [66]. However, IL-10 in Tregs was shown to be dispensable in the regulation of the development of a spontaneous mouse colitis model where IL-10 was ablated in FoxP3^+^ Tregs [67]. Growing evidence regarding the biology of IL-10 in T cells suggests that Th1 and Th17 cells self-limit their responsiveness and reduce their inflammatory function via IL-10 expression, suggesting a crucial role of IL-10 in T cells to restrain their effector function [68]. IL-22, an IL-10 family cytokine, plays an important role in maintaining mucosal barrier function and in the pathogenesis of IBD [69,70]. In the intestinal mucosa of IBD patients, IL-22-producing CD4^+^ T cells appeared to be decreased in actively inflamed tissues [71,72], and cluster analysis highlighted that the IL-22-producing T cell population should be considered independently from the Th17 and Th1 subsets [73]. Furthermore, Th22 and IL-22^+^ Th1 populations were decreased in UC compared to CD and heathy controls [72]. Since micro-environmental cytokines are critical for regulation of plasticity between Th lineages to reprogram their differentiated states [74], it was demonstrated that Th17 cells could be converted into either regulatory T cells in the presence of TGF-β or Th1 cells in response to inflammatory cytokine stimulation (e.g., IL-12 and IL-23) [75]. Understanding the cytokine-mediated regulation of plasticity between Th subsets is essential to develop novel biological therapies for IBD treatment by enabling specific Th cell polarizing or reprograming events.

## 2. Self-Limitation of T Helper Cell Pathogenicity through IL-10

Based on patterns of cytokine secretion, subsets of the same lineage may express different effector cytokines or acquire features of different fates to secrete common cytokines, such as IL-10, resulting in heterogeneity of the Th population. In Th1, Th2, and Th17 subsets, different STATs mediate distinct differentiation programs to commonly induce IL-10 expression; TGF-β is also considered to be an essential driver of IL-10 expression [76,77,78,79,80,81]. Either c-Maf or B lymphocyte-induced maturation protein-1 (Blimp-1) can broadly promote IL-10 expression in both effector and regulatory T cell subsets [76,78,79,82,83,84,85,86,87,88,89,90]. Th1 and Th17 cells eventually express IL-10 as a negative feedback mechanism to regulate the immune response [76,91]. IL-10 expression in Th1 cells is under TCR stimulation together with IL-12, while the production of IL-10 driven by TGF-β and IL-6 is an intricate part of Th17 cell differentiation [76,77,78,92]. IL-27 is critical for the development of IL- 10^+^ IFN-γ^+^ Th1 cells to prevent immune pathology in mice infected with *T. gondii* [93,94]. The delta-like-4/Notch axis together with IL-12 or IL-27 enhance IL-10 production and anti-inflammatory capacity in IFN-γ-producing Th1 cells [95,96]. Taken together, IL-10 induction in Th lineages may represent plasticity of several T helper cell differentiation pathways. Accordingly, better understanding of the extrinsic and intrinsic signals required to reprogram Th lineages toward a suppressive phenotype may have important therapeutic applications in the maintenance of self-tolerance and tissue homeostasis. This section could be divided by subheadings and should provide a concise and precise description of the experimental results, their interpretations, and the experimental conclusions that can be drawn.

### 2.1. Non-Pathogenic or Anti-Inflammatory IL-10 Producing Th1 Cells and Plasticity toward Tr1 Cells

**Figure 2:**
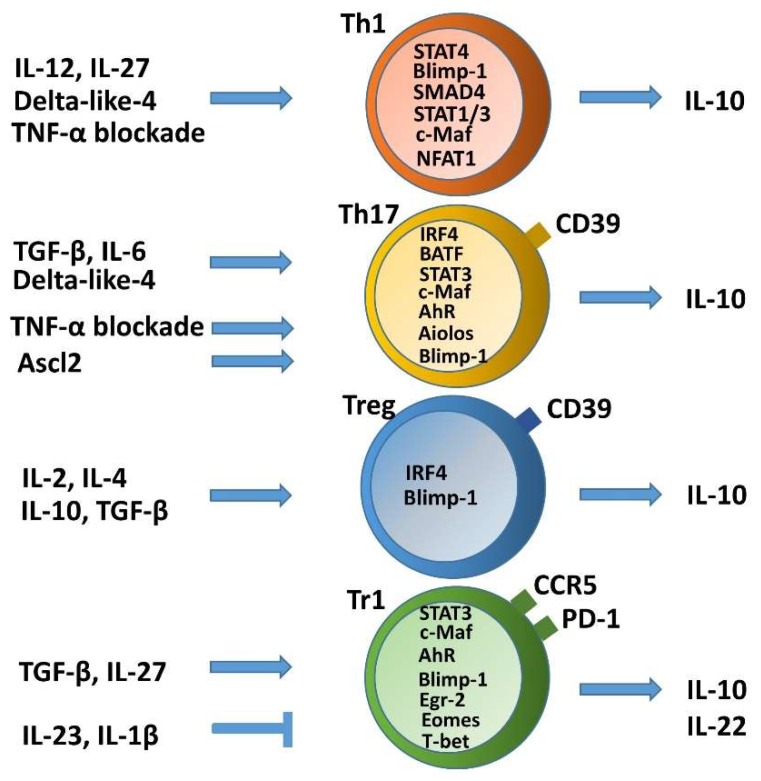
IL-10 expression in T helper lineages represents plasticity of several T helper cell differentiation pathways. Different T cell subsets secrete IL-10 when stimulated by a variety of cytokines. In particular, IL-27 and TGF-β broadly induce IL-10 production from various T cell subsets.

Differentiation of non-Foxp3-expressing Tr1 cells (characterized as IL-10^+^IFN-γ^+^ double producers) is regulated by the heterodimeric cytokine IL-27, consisting of EBI3 and p28 subunits,; these Tr1 cells execute their suppressor functions by secreting IL-10 through a c-Maf/Ahr-dependent mechanism or activation of STAT3 and Egr-2 in a Blimp1-dependent manner [88,92]. Blimp-1 expression is critical for IL-10 production in Th1 cells and dependent on STAT4, downstream of IL-12 signaling. IL-27 also promotes Blimp-1-dependent IL-10 production in Th1 cells by signaling through STAT1/3 [79]. Furthermore, downstream of T-bet and IL-27, Eomos is expressed and cooperates with Blimp-1 to transcriptionally activate IL-10 expression in human and murine Tr1 cells [43,97]. Moreover, IL-10/IFN-γ co-expressing CD4^+^ T cells induced by tolerogenic dendritic cells present a strong regulatory profile and display potent suppressive capacity over Th1-mediated activation [98]. Therefore, IL-10 induction may depend on both the cytokine environment and the molecular context, implying that Tr1 cells exhibit plasticity. Intestinal IFN-γ^+^ Tr1 cells, which are co-expressed with C-C chemokine receptor type 5 (CCR5), and programmed cell death protein 1 (PD-1), with immunosuppressive properties were first identified in human and mouse subjects with IBD (Figure 2). Selective downregulation of IL-10 expression in intestinal IFN-γ^+^ Tr1 cells, but not Th cells or CD25^+^ Treg cells, was observed in patients with IBD; possible regulation by pro-inflammatory cytokines, IL-1β and IL-23 suggested a critical role of IFN-γ^+^ Tr1 cells in control of intestinal inflammation [99]. Tr1 cells isolated from healthy individuals and patients with CD or UC were also found to secrete IL-22 to promote barrier function of human intestinal epithelial cells [100]. A recent study demonstrated that children with IBD in both CD and UC groups presented increased Tr1 cells at diagnosis, which decreased at follow-up compared to diagnosis. This was particularly apparent in UC, indicating that compensative upregulation of Tr1 is insufficient to counteract the inflammation [101]. A therapeutic strategy using single-chain human IL-27 suppressed several inflammatory cytokines, including IL-17, but promoted IL-10 secretion in a TNBS-induced mouse colitis [102]. In accordance with findings showing that therapeutic antibodies blocking TNF-α enhanced IL-10 production by all effector T cell subsets in vitro [103,104], targeting tumor necrosis factor receptor 1 assembly was shown to suppress Th1 and Th17 effector phenotypes by increasing the frequency of IL-10-producing populations and the levels of IL-10 in Th1 and Th17 cells in a T cell-specific, Blimp-1-deficiency-mediated colitis model [105]. Development of a Tr1 cell-based therapy for intestinal inflammation may suppress both proliferation of effector T cells and production of pro-inflammatory cytokines, leading lead to long-lasting remission and a possible cure for IBD.

### 2.2. Non-Pathogenic or Anti-Inflammatory IL-10-Producing Th17 Cells

Growing evidence suggests that the Th17 subset is a heterogeneous population composed of pro-inflammatory subsets acting as pathogenic effectors through the production of IL-17 and anti-inflammatory subsets exhibiting regulatory properties with co-production of the anti-inflammatory cytokine IL-10, a subset referred to as regulatory Th17 cells [106,107]. TGF-β and IL-6-mediated production of IL-10 represent part of Th17 cell differentiation program (Figure 2) [78,92]. In IL-10-producing Th17 cells, an immunoregulatory and tissue-residency program including IL-10 expression is governed by selective upregulation of c-Maf via its binding to a set of enhancer-like regions [108]. TNF-α blockade was shown to promote IL-10 production in human Th17 cells characterized by expression of Aiolos, an Ikaros family member, which binds to conserved regions in the IL10 locus in Th17 subsets [103,104]. Th17 cells were shown to differentiate into Tr1 cells during resolution of intestinal inflammation. Moreover, a previous report described that Th17 cells expressing CD39 ectonucleotidase produced IL-10 and were less pathogenic than CD39-negative Th17 cells in a T cell-transfer colitis model in Rag^-/-^ mice. Importantly, CD39 expression by Th17 cells hydrolyzes ATP to block ATP-induced cell death and is crucial for IL-10 production, suggesting a critical role for purinergic signaling as a regulator of Th17 cell plasticity in intestinal inflammation [109]. Furthermore, achaete-scute complex homologue 2 (Ascl2) inhibits Th17 cell differentiation and restrains their pathogenicity via promoting Blimp-1-dependent IL-10 production, whereas Ascl2 was shown to be significantly downregulated in human IBD and experimental colitis [110]. Similarly, through facilitating Blimp-1-mediated IL-10 production in Th17 cells, Sauchinone (SAU), a key bioactive lignin isolated from the roots of *Saururus chinensis*, ameliorated TNBS-induced colitis and modulated inflammatory responses in mucosal tissues and peripheral blood CD4^+^ T cells in IBD patients [111]. These recent findings extend our view of IL-10 secretion as a negative feedback mechanism in Th17 cells, indicating that this could serve as a novel therapeutic target of IBD.

## 3. Heterogeneity and Plasticity of T Helper Cells in Inflammatory Bowel Disease (IBD)

T cell lineage commitment to a single fixed Th phenotype has been challenged by increasing evidence of functional plasticity within CD4^+^ T cell subsets with the ability to change between helper phenotypes, or helper and regulatory functions. The capacity for T cell lineages to switch between patterns of cytokine secretion and effector functions from one lineage to another under tissue environmental cues is thought to be reprogrammed by the expression of transcription factors characteristic of opposing lineages. Notably, recent evidence on conversion among polarized T helper cells highlighted the significance of Th cell plasticity and transdifferentiation in the pathogenesis of IBD [112,113,114,115,116].

### 3.1. Th1 Plasticity

Although the well-defined Th1 lineages are thought of as terminally committed effector cells which exhibit low levels of plasticity in changing their phenotype, fully differentiated myelin oligodendrocyte glycoprotein (MOG)-specific Th1 clones are still capable of producing IL-17 upon superantigen stimulation [117]. In addition, human Th1 cells exposed to Th17-polarizing conditions undergo lineage conversion and exhibit a Th1/17 phenotype by upregulating IL-17 upon enforced expression of *RORC2* but without losing expression of IFN-γ or *TBX21* [118]. Retinoic acid (RA) signaling was reported to be important for limiting Th1 cell conversion into Th17 effector cells and for preventing pathogenic Th17 responses in vivo through maintaining stable expression of Th1 lineage-specific genes, as well as repressing genes that instruct Th17-cell fate [119]. IFN-γ-producing Th1 effector cells specific for an intestinal microbial antigen, CBir1 flagellin, induced colitis in Rag^-/-^ mice after adoptive transfer and acquired IL-17-producing capacities in the gut. Th1 conversion into Th17 cells is regulated by TGF-β and IL-6, and mediated by TGF-β induction of Runx1. TGF-β enhances accessibility of Runx1 binding to RORγt and IL-17 promoters in Th1 cells, supporting a possible mechanism for Th1 to Th17 cell transdifferentiation under inflammatory conditions in the intestine [120]. Another report uncovered a small ubiquitin-related modifier (SUMO)ylation-based mechanism that controls Th1/Th17 plasticity, SUMOylation-deficient Wiskott-Aldrich syndrome protein (WASp), which favors ectopic development of the Th17-like phenotype under Th1-skewing conditions, while SUMOylated nuclear-WASp serves as a transcriptional promoter-specific coactivator, which is essential in Th1 gene transcription [121]. However, IFN-γ-producing cells tend not to develop into Tregs and inhibit the generation of Tregs in vivo, suggesting limited plasticity of Th1 populations to Tregs [122]. Nevertheless, upon short-term culture in vitro, effector CD4^+^CD25^-^CD127^+^ T cells from human peripheral blood could convert into T cells with regulatory activity while expressing a panel of common Treg markers, including FOXP3, CD25, GITR, HLA-DR, and CTLA-4, in parallel with the Th1-specific transcription factor T-bet and concomitantly secreting IFN-γ [123]. These data reveal a unique regulatory network governing maintenance and plasticity of Th1-cell fate and define an additional pathway for the development of Th17 cells.

### 3.2. Th17 and Treg Imbalance Paradigms

**Figure 3:**
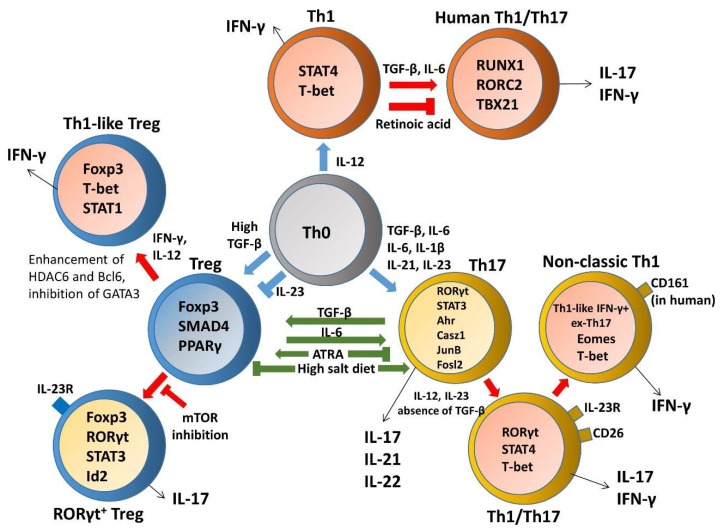
The plasticity and transdifferentiation of Th1, Th17, and Treg cells. Cytokine networks influencing T cell development are indicated in blue arrows and extrinsic signals that control plasticity are shown in red arrows. Cytokine production by distinct T helper cells is indicated in black arrows and components involved in regulating Th17/Treg cell imbalance paradigms are indicated in green arrows.

A loss of homeostasis between regulatory and inflammatory CD4^+^ T cell populations results in subsequent intestinal tissue damage, while increased Th1, Th2, Th9, and Th17 responses and reduced Treg and Tr1 responses were suggested to play a role in IBD pathophysiology [115]. Although Th17 and Treg cells are two distinct T cell subsets with opposite effects on immune functions, the reciprocal differentiation of Th17 cells and Treg cells is closely related and plays a critical role in both the pathogenesis and resolution of IBD. Tregs were capable of suppressing Th17-mediated colitis in an adoptive transfer model of colitis [124]. Decreased Tregs, increased Th17 cells, and a significant decrease in the Treg/Th17 ratio were observed in the peripheral blood of IBD and pediatric IBD patients; this reduced ratio is associated with serum IL-6 and IL-23 levels, suggesting that the Th17/Treg cell balance plays an important role in the development and maintenance of inflammation [125,126,127]. In particular, disordered regulation of Th17 and Treg cells in IBD is caused by a disruption in the chemokines and, consequently, their cognate receptors, thereby disturbing mucosal homeostasis [35,128]. IL-6 is a proinflammatory cytokine favoring Th17 differentiation, whereas SMAD4 is only involved in Foxp3 upregulation. TGF-β signaling is required for both Foxp3 and IL-17 induction. However, IL-6 is crucial for the balance between pathogenic Th17 and protective Treg by overcoming the suppressive effect of Foxp3 (Figure 3). Moreover, IL-6 and IL-1 induce genetic reprogramming in Treg cells. Foxp3 antagonizes RORγt to inhibit Th17 differentiation while STAT3 downregulates Foxp3 expression and is critical for IL-17 expression in Treg cells [129]. Further, environmental cues including the gut microbita and dietary components are involved in regulating Th17/Treg cell imbalance paradigms. All-trans retinoic acid (ATRA), the active derivative of vitamin A, inhibits differentiation of Th17 cells and also induces Treg differentiation in colon biopsies of patients with UC in vitro and in TNBS-induced murine colitis, restoring the Th17/Treg balance [130]. A high salt diet stimulated the intestinal Th17 response but inhibited the function of Treg cells in TNBS-induced mice colitis [131]. Segmented filamentous bacteria modulate the homeostatic plasticity of T helper and T regulatory cells and coordinate the intestinal T cell profile [132]. A plethora of therapeutic strategies for IBD regulation demonstrated that DSS-induced mouse colitis was ameliorated via regulating the balance between Th17 and Treg cell to control intestinal inflammation [133,134,135,136]. Moreover, impaired delivery of IL-15 to CD4^+^ T cells and CD4^+^ T cells deprived of IL-15 can trigger intestinal inflammation by accumulation of Th1/Th17 cells and fine-tune the balance between Treg and Th17 cells by downmodulating Foxp3 expression and enhancing RORγt expression [137]. Furthermore, growing evidence reports remarkable plasticity between the Th17 and Treg cells reflected in the capacity of differentiated effectors cells to be reprogrammed among Th17 and Treg lineages [138]. A crucial need is emerging to understand the complexity of the specificity and plasticity of effector Th17 and suppressive Treg cells, which are central to the pathogenesis of IBD.

### 3.3. Th17 Plasticity

In contrast to the classic Th1 and Th2 cells, studies of Th17 differentiation elucidated that developmental flexibility and heterogeneity allows them to acquire cytotoxic activity or regulatory phenotype in the periphery in response to additional environmental challenges within the tissue [139]. Since the initial finding that TGF-β is required for both Th17 and Treg cell differentiation, plasticity between Treg and Th17 cell programs was shown to be reciprocally regulated. Subsequent findings revealed that differentiated Th17 subsets displayed a high grade of plasticity to produce predominantly IFN-γ in terminal differentiation, which is not an endpoint of T cell development, implying substantial overlap with conventional Th1 cells [140,141]. It became apparent that cytokine signals and transcriptional factors determine the fate of Th17 cell plasticity, leading them to either fully acquire their pathogenicity or divert toward a regulatory fate. Evidence from animal and human IBD studies highlighted an important role of T cell plasticity in the regulation of mucosal homeostasis and inflammation, such as Th1–Th17 and Th17–Treg axes. Moreover, increased numbers of transdifferentiated T cell populations developed in IBD patients displayed a high grade of plasticity [142,143,144]. Taken together, deeper insights into plasticity in inflammatory conditions could contribute to the design of therapeutic strategies for IBD by reprogramming the fate of Th17 in the gut.

#### 3.3.1. Th17–Th1 Plasticity and Transdifferentiation

Th17 cells display significant plasticity and convert to Th1-like cells under pathogenic conditions. With exposure to Th1-polarizing conditions, in vivo-differentiated human Th17 cells upregulated *TBX21* and IFN-γ without loss of IL-17 or RORC expression and resulted in a Th1/17 phenotype [118]. Accumulating evidence shows that Th17 cells in inflammatory conditions are highly plastic and can transdifferentiate to IL-17/IFN-γ double-producing cells and Th1-like IFN-γ^+^ ex-Th17 lymphocytes (non-classic Th1), which seem to be more pathogenic than the unshifted cells in animal models of IBD [145,146]. Remarkable features of the pathogenic Th17 cell phenotype included co-expression of RORγt and T-bet and high production of both IL-17 and IFN-γ, whereas non-classic Th1 cells, characterized by extinguished IL-17 expression and acquired expression of IFN-γ, produced increased amounts of proinflammatory cytokines and were more polyfunctional in terms of cytokine production than either Th1 or bona fide Th17 cells [146]. It was supported by the available human data that the percentages of Th17 and Th1/Th17 cells were increased in patients with IBD and a subpopulation of Th17 cells sharing a Th1 signature with high expression of T-bet, CD26, and IL-22 were shown to be involved in mucosal inflammation of CD and UC patients [26,147]. In addition, a larger proportion of commensal-specific CD4^+^ T cells from patients with CD exhibited a Th17 phenotype or produced Th1 and Th17 cytokines compared with T cells from controls [148]. In humans, distinctive features of Th17-derived Th1 cells (non-classic Th1) appear to be the expressions of CD161 and interleukin-4-induced gene 1 (IL4I1), which distinguish between non-classic Th1 and classic Th1 cells and are virtually absent in classic Th1 cells [149,150]. Additionally, CD4^+^CD161^+^ T cells with Th17, Th17/Th1, and Th1 phenotypes accumulate in CD perianal fistulas rather than in the peripheral blood [151]. On the contrary, murine Th17 cells do not express CD161. This Th17 to Th1 cell plasticity was also confirmed in mice by utilization of an IL-17 fate-mapping mouse strain. Th17 cells rapidly lost their IL-17 expression and were converted into Th1 cells independently of IL-7 signaling after transfer into a lymphopenic host [152]. Further evidence showed that Th17 cells generated either in vitro or in vivo represented a transient phenotype that tended to convert into IFN-γ-producing cells and appeared to be pathogenic upon adoptive transfer of IL-17F-reporter-positive Th17 cells to Rag-deficient mice [153].

The stability of the Th1 phenotype in Th17 cells is under control of molecular machinery governed by cytokine networks and transcription factors. In the absence of TGF-β, both IL-12 and IL-23 suppress IL-17 and enhance IFN-γ production from Th17 cells in a STAT4- and T-bet-dependent manner [115]. IL-12 was reported to inhibit the expression of the lineage-specific transcription factors Foxp3 and RORγt during the development of Tregs and Th17 cells, respectively, and increase expression of T-bet and production of IFN-γ, suggesting IL-12 skews the TGF-β-dependent differentiation programs into a Th1-like phenotype [154]. Downstream IL-12 signaling removed H3K27 trimethylation modifications at *Tbx21*, leading to enhanced T-bet expression and induced silencing of the *Rorc* gene via a STAT4/T-bet dependent pathway in ex vivo- and in vitro-generated Th17 cells [155,156]. Moreover, IL-23 deprivation converts IL-17 producing cells to IFN-γ secreting Th1 phenotype [157]. The colitogenic potential of IFN-γ-producing Th17 was verified by a study focusing on IFN-γ-deficient Th17 cells, which retained an IL-17A^+^ phenotype and were unable to induce colitis in a Th17-transfer colitis model, while development of disease required the transition of Th17 precursors to Th1-like cells depending on the expression of both STAT4 and T-bet [158]. Th17 cells expressing IL-23R, but not IL-12R, were predisposed to upregulation of the Th1 program in inflamed intestines from a bacteria-induced typhlocolitis mouse model and subsequently contributed to intestinal pathology by switching phenotype, transitioning via an IL-17/IFN-γ double-producing stage to become IFN-γ^+^ ex-Th17 cells [159]. Moreover, *Tbx21* deficiency in IL-17-producing or Rag1-expressing cells had no effect on the generation of IL-17/IFN-γ double-producing cells, but led to a significant absence of Th1-like IFN-γ^+^ ex-Th17 cells in Helicobacter hepaticus-induced intestinal inflammation, however, the degree of mucosal inflammation was indistinguishable from that observed in control mice [160]. Studies in humans subsequently demonstrated that IL-12, but not IL-23, enhanced IFN-γ expression in CCR6^+^CXCR3^-^RORγ^+^Tbet^-^CD4^+^ Th17 effector memory T cells and switched their phenotype to become Th1-like IFN-γ^+^ ex-Th17 cells in mesenteric lymph nodes (mLNs) of both CD and UC patients, suggesting that Th17 plasticity occurs in mLNs before their recruitment to the inflamed colon [161]. The activator protein-1 (AP-1) factor JunB was also reported to be an essential regulator of Th17 cell identity and a promoter of Th17 cell subset stability in vivo, while JunB activates the expression of Th17 lineage-specific genes and restrains alternative Th1 and regulatory T cell potential during Th17 cell induction, indicating that sustained JunB expression is essential to limit plasticity in differentiated Th17 cells [162]. A new Th plasticity regulator, Casz1, was identified to limit repressive histone marks and enable acquisition of permissive histone marks at *Rorc*, *Il17a*, *Ahr*, and *Runx1* loci under Th17 differentiation conditions. Importantly, in mucosal Candida infection, loss of Casz1 caused a marked diminution in IFN-γ^+^IL-17A^+^ double-positive inflammatory Th17 cells in tissues in vivo [163]. Since signaling via the kinase complex mammalian target of rapamycin complex 1 (mTORC1) tightly controls the fate of Th17 cells, they fail to develop into Th1-like cells upon loss of mTORC1 activity [164]. A recent report demonstrated that Eomes was critical in the stability of non-classic Th1 phenotype via promoting IFN-γ secretion while inhibiting the expression of RORγt and IL-17 (Figure 3). Since non-classic Th1 cells promote the generation of IFN-γ^+^ GM-CSF^+^ cells that were described to be pathogenic in IBD, Eomes promoted their pathogenic potential in a T cell-transfer mouse colitis model [42].

#### 3.3.2. Th17-Treg Plasticity

The transdifferentiation of Th17 into regulatory T cells changes their signature transcriptional profile and acquires features of potent regulatory capacity to contribute to the resolution of inflammation. The Th17 lineage shows functional plasticity in the gut and conversion into regulatory T cells, which can co-express Foxp3, a signature transcription factor of Tregs that plays an important role in the regulation of the intestinal immune responses [142,143,165]. A previous study established a critical role for human myeloid-derived suppressor cells (MDSCs) in converting monocyte-induced Th17 cells into Foxp3-expressing Tregs through MDSC-derived, membrane-bound TGF-β and a retinoic acid-dependent mechanism [166]. A predictive model validated by computational and experimental modeling identified FOS-like antigen 2 (Fosl2) as a key determinant of cellular plasticity in Th17 cells. Fosl2-deficient mouse CD4^+^ T cells yielded IL-17-producing cells, which co-expressed Foxp3 under Th17-polarizing conditions and enabled IFN-γ production in Th17 cells under Th1-skewing conditions [167]. Further reports showed that a critical role for peroxisome proliferator-activated receptor gamma (PPARγ) was predicted in modulating plasticity between Th17 and Tregs by utilization of experimental approaches and computational modeling (Figure 3). Pharmacological activation of PPARγ in a T cell-transfer colitis model resulted in increased colonic Tregs and decreased Th17 cells in the gut mucosa, whereas deletion of PPARγ in CD4^+^ T cells impaired mucosal Tregs and enhanced colitogenic Th17 responses in mice with CD4^+^ T cell-induced colitis [168].

#### 3.3.3. Th17-Tfh Plasticity

Follicular helper T (Tfh) cells are a T helper subset specialized to regulate antibody response. Intestinal Th17 cells were shown to converge toward a Tfh cell phenotype in Peyer’s patches, which are essential lymphoid organs for the generation of T cell-dependent immunoglobulin A (IgA) for intestinal homeostasis, independent of IL-23 for their plasticity. Mice deficient in Th17 cells failed to generate antigen-specific IgA responses after immunization with cholera toxin, providing evidence that Th17 cells are a crucial subset required for inducing the development of high-affinity, T cell-dependent, IgA-producing, germinal center B cells [169].

### 3.4. Treg Plasticity

Recent studies suggested FoxP3^+^ Treg cells are a heterogeneous population consisting of a committed Treg lineage and an uncommitted subpopulation with a high grade of plasticity, allowing them to their cell fate to various effector T cell phenotypes as a functional adaptation to inflammation and as a critical factor for autoimmune disease. However, whether post-conversion Tregs maintain stable Foxp3 expression as well as suppressive function or lose Foxp3 expression remains under debate [170,171,172].

#### 3.4.1. Treg–Th17 Plasticity and Transdifferentiation

Besides suppressive function, plasticity enables a fraction of Tregs to differentiate into IL-17-producing Th17 cells, a conversion associated with autoimmune inflammation [173]. The fact that Treg cells induce themselves to convert into Th17 cells in the presence of IL-6 and in the absence of TGF-β upon stimulation suggest a potential plasticity of Treg cells to transdifferentiate into pathogenic Th17 cells during mucosal inflammation in patients with IBD [174]. IL-17^+^ FoxP3^+^ T cells, a novel crossover immune cell population, were identified in inflamed intestinal mucosa of patients with CD and showed potent suppressor activity in vitro [175]. Similarly, an increased prevalence of IL-17 and Foxp3 double-expressing CD4^+^ T cells that co-expressed RORγt and Foxp3 was also observed in PBMCs of patients with CD and UC. However, the authors suggested that increased IL-17^+^ FoxP3^+^ cells in the disease state may give rise to the low suppressive ability of Treg cells isolated from patients with IBD, indicating that Treg cells from patients with IBD could modify their phenotypes, leading to inflammation [176]. Additionally, IBD patients display increased crossover populations in Treg cells toward Th2 and Th17 in the intestinal lamina propria, a phenomenon associated with the clinical disease score of IBD [72]. On one hand, the frequency of Foxp3^+^ RORγt^+^ cells in a Foxp3^+^ cell population was increased and associated with enhanced IL-23R expression level in colon mucosa during DSS-induced acute and chronic colitis, suggesting the importance of IL-23 signaling related to the instability of Treg cells in the development of IBD [177]. On the other hand, RORγt^+^ Treg cells decreased after transfer of IBD microbiota into germ-free mice, while colonization with IBD microbiotas exacerbated disease in a T cell-transfer mouse model, supporting a beneficial role of RORγt^+^ Treg cells in IBD [178]. The gut microenvironment facilitates the reprogramming of Treg cells into effector T cells and promotes host immunity. Treg cells lose Foxp3 expression and predominantly produce IL-17 in mesenteric sites. This reprogramming of Treg cells can be modulated by mTOR inhibition with the immunosuppressive drug rapamycin to stabilize Foxp3 expression [179]. The ability of Treg cells to reprogram themselves is thought to be regulated by mechanisms that control the expression of transcription factors. STAT3 downregulates Foxp3 expression while STAT3 and RORγt are required for IL-17 expression in Treg cells [129]. The enhanced expression of the transcription regulator Id2 induced by pro-inflammatory cytokines drives the generation of pathogenic ex-Foxp3 Th17 cells from Tregs by sequestration of the transcription activator E2A, subsequently reducing the expression of Foxp3 and leading to the induction of Th17-related cytokines (Figure 3) [180].

#### 3.4.2. Treg-Th1 Plasticity and Transdifferentiation

Both murine and human Foxp3^+^ Treg were recently shown to express T-bet and IFN-γ, characteristic of the Th1 effector cell phenotype. Co-culture of naïve human CD4^+^ T cells with high numbers of CD40-activated allogeneic B cells resulted in preferential differentiation into alloantigen-specific CD4^+^CD25^+^ Tregs, which exhibit potent suppression functions and express T-bet, IFN-γ, and CXCR3, the features of Th1 effector cells [181]. In addition, Tregs isolated from the lamina propria of active, but not inactive, IBD patients or uninflamed controls expressed T-bet and IFN-γ. As observed in human IBD, Th1-like Tregs were upregulated in the inflamed lamina propria of DSS-treated mice [182]. In response to pathological cues, Treg cells acquire a Th1-like phenotype by upregulating T-bet expression upon IFN-γ stimulation in a STAT1-dependent manner, while T-bet^+^ Foxp3^+^ Tregs retain their suppressive ability and contribute to resolution of type I inflammation. Subsequent exposure to IL-12 promotes Tregs to express T-bet and release IFN-γ. These IFN-γ-producing Tregs either contribute to the resolution of type I inflammation (Th1-suppressing) or lose suppressive function and fail to efficiently control autoimmune responses (Th1-like Tregs). The molecular mechanisms underlying Th1-like Treg differentiation demonstrated that enhanced AKT phosphorylation led to increased FOXO1/3 nuclear export, which activated downstream signaling to trigger T-bet and IFN-γ gene expression [183]. Further studies indicated that Foxp3^+^ Treg cells specific for CBir1 flagellin, an immunodominant microbiota antigen, were able to convert into IFN-γ^+^ Th1 cells, IL-17^+^ Th17 cells, and Foxp3^+^ T cells co-expressing IFN-γ and/or IL-17 in the intestine. Blocking IL-12 with a neutralizing antibody inhibited Treg conversion to Th1 and IFN-γ^+^ Foxp3^+^ T cells in the intestines of mouse recipients of Treg cells, suggesting that innate cells are required to produce IL-12 during the transition of Treg cells toward the Th1-like phenotype (Figure 3). Moreover, IFN-γ^+^ Foxp3^+^ T cells displayed potent suppressive capacity to suppress proliferation of naïve T cells in vitro and inhibit induction of colitis by microbiota antigen-specific T cells [184]. On the contrary, Th1-like Tregs may contribute to inflammation rather than suppress it. Indeed, Treg-specific T-bet-knockout mice developed milder colitis after DSS treatment and displayed reduced expression of IFN-γ among lamina propria CD4^+^ T cells not restricted to T-bet-deficient Tregs, but this response also involved conventional T cells, indicating that the Th1 immune response depends on the presence of Th1-like Tregs. Meanwhile, T-bet deficiency did not affect Treg suppressive capacity either in vitro or in vivo in the adoptive transfer model of colitis. Therefore, T-bet expression in Tregs sustained the early phase of the Th1-mediated inflammatory response in the gut [182]. Since optimal FOXP3 expression highly depends on hypomethylation of the *FOXP3* gene, treatment of the DNA methyltransferase inhibitor induced plasticity in human Tregs by increasing IFN-γ expression and induction of a Th1 phenotype while maintaining the suppressive function [185]. While EZH2 is recruited to the Foxp3^+^ promoter and its targets in Treg cells, EZH2-deficient Foxp3^+^ T cells lack a regulatory phenotype in vitro and secrete proinflammatory cytokines. Moreover, EZH2^Δ/Δ^Foxp3^+^ mice developed spontaneous inflammatory bowel disease. Gene network analysis in isolated intestinal CD4^+^ T cells from patients with CD demonstrated that these CD4^+^ T cells displayed a Th1/Th17-like phenotype with enrichment of gene targets shared by FOXP3 and EZH2, suggesting that deregulation of FOXP3/EZH2 T cell gene networks in Crohn’s disease could contribute to the underlying intestinal inflammation [186]. Other important regulators of Treg plasticity include enhancement of HDAC6 and Bcl6, as well as inhibition of GATA3 [187].

## 4. Conclusions

Dysregulation of the balance between effector and regulatory T cell phenotypes is critical for disruption of intestinal immune homeostasis leading to autoimmune disease. The plasticity and transdifferentiation among CD4^+^ T cell lineages in the gut is strongly associated with IBD pathogenicity and controlled by genetic regulation of gene expression through the concerted action of cytokines, transcription factors, and epigenetic regulators. According to the stimuli received, Th1 and Th17 cells show high plasticity toward a regulatory phenotype via alteration of the cytokine program to secrete IL-10, an important anti-inflammatory cytokine involved in the control of the mucosal immune response in IBD. Th17 and Treg cells exhibit both instability regarding abrogation of their signature cytokine productions and flexibility in their expression of cytokines of other lineages. In response to changing environmental cues, Th17 and Treg cells acquire global genetic reprogramming to drive their conversion from one T helper cell type to another. Either plasticity or lineage conversion of T helper cell populations can shape adaptive immunity to prevent or promote pathogenic immune responses. Expanding our understanding of T cell diversity could offer new insight into the disease-related heterogeneity and plasticity of T helper cell responses, and the modulation of homeostatic plasticity of T helper and T regulatory cells may provide therapeutic opportunities for IBD.
